# Gut Microbiota Provide Co‐Existing Strategies for Two Species of Symmetrically Distributed Rodents in Competition for Food

**DOI:** 10.1002/ece3.72290

**Published:** 2025-10-20

**Authors:** Yue Ren, Mengfan Tao, Guangtong Guo, Kuiyou Chen, Xinsheng Pu, Yu Hou, Xin'gen Yang

**Affiliations:** ^1^ College of Plant Protection Shanxi Agricultural University Taiyuan China; ^2^ Shanxi Key Laboratory of Integrated Pest Management in Agriculture Shanxi Agricultural University Taiyuan China

**Keywords:** *Apodemus agrarius*, coexist, *Cricetulus longicaudatus*, gut microbiota, interspecific competition

## Abstract

Gut microbiota provides an effective strategy for sympatric proximal species to coexist in interspecific competition. In the present study, 16S rRNA gene sequencing was used to investigate the gut microbial of the *Cricetulus longicaudatus* and *Apodemus agrarius* , which are two species distributed in the same domain, under natural ambient and varying dietary situations. Our data revealed that there were significant differences in gut microbial structure and diversity between the two species. Specifically, the *C. longicaudatus* demonstrated high alpha diversity and an abundance of *Lactobacillus*, whereas *A. agrarius* showed substantial enrichment of Verrucomicrobiota. Wild *C. longicaudatus* had a more complex co‐occurrence network, with a low level of positive correlation rate; however, after being fed various diets, the network structure was simplified, and the positive correlation rate increased. On the contrary, wild *A. agrarius* had a simple co‐occurrence network, with a high level of positive correlation rate; after exposure to different diets, the network structure became more complex, accompanied by a decrease in the positive correlation rate. Our results also revealed differences in dietary adaptation between the two species. *C. longicaudatus* exhibited a greater microbial adaptability under a high‐fat and high‐fiber diet than *A. agrarius* , as indicated by a significant rise in the Firmicutes/Bacteroidetes ratio. While *A. agrarius* demonstrated reduced adaptation to dietary changes, it had a stronger ability to adapt to a high‐fat diet than to a high‐fiber diet. Finally, our data revealed significant alterations in carbohydrate metabolism pathways between the two species. This study provides new insights into how the gut microbiota of symmetrically distributed rodents provides effective survival strategies for species in the face of competition.

## Graphical abstract

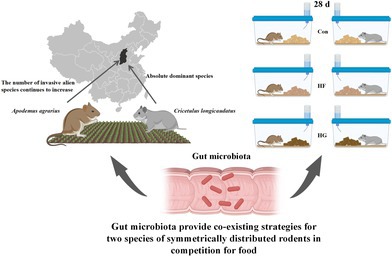

## 1 Introduction

Sympatric species may require the same food, habitats, and engage in the same reproductive periods; these overlaps tend to result in competition. When two closely related species live in the same environment, they will compete with each other. This can eventually lead to two outcomes: One species will dominate, and the other will be pushed to the edges, which could lead to its extinction (Jaeger 1970); or the two will show niche differentiation, which includes differences in habitat, food resources, and activity time (Coyte and Rakoff‐Nahoum 2019). Three strategies are available for two closely related species in sympatric distributions to mitigate competition: first, they may circumvent competition by inhabiting adjacent yet distinct geographic areas (Hairston 1949); second, they can coexist while utilizing different habitat types to prevent rivalry (Dumas 1956); and third, even closely related species sharing a habitat can evade competition by altering their utilization of environmental and food resources (Jaeger 1970). Researchers have found that *Rattus flavipectus* and *Niviventor confucianus* store more food in a concentrated way when they are in a competitive environment. *N. confucianus* also changes from scatter hoarding to concentrated storage. This phenomenon diminishes the availability of seed resources, thus reducing direct competition with rivals (Zhang et al. 2013). Animals not only optimize food utilization of resources in their behaviors but also exhibit extraordinary adaptability in the utilization and absorption of nutrients during digestion (Wang et al. 2003).

Gut microbiota play a crucial role in facilitating the host's adaptability to diverse nutritional resources (Lu et al. 2024). They can enhance the host's efficiency in food digestion and nutrient absorption, optimize the host's energy conversion rate, and bolster the host's resistance, allowing it to maintain essential physiological activities even in adverse conditions (Gao et al. 2024; Ross et al. 2024). Mammals contain trillions of gut microorganisms that are essential for food digestion, supporting host health, and aiding the host in adapting to varying environments (Clemente et al. 2012). Herbivores and their gut microbiota share a symbiotic relationship that has facilitated their adaptation to specific settings across prolonged evolutionary periods (Anderson and Briske 1995). The gut microbiome has considerable plasticity in response to extreme dietary scarcity. Research on the adaptation of animals revealed that, despite a relatively low diversity in the gut microbiome, its functioning exhibits major adaptation, helping the host to survive in regions with limited food supplies (Kohi 2020; Hernández et al. 2024; Yang et al. 2024). This indicates that the gut microbiome not only facilitates short‐term energy acquisition but also serves as the biological foundation for the long‐term evolution of species (Vadstein et al. 1993). Specific gut microorganisms may aid the host in acquiring the energy necessary for survival in extreme cold and food‐deficient environments by digesting particular carbon sources (Qin et al. 2020). Studies on mammals have shown that different species could increase their competitive advantage for food resources via the selective adaptation of their microbiomes. For example, the gut microbiota of the greater kudu ( *Tragelaphus strepsiceros* ) can totally decompose complex plant sources, thereby extracting more energy, which enables their survival during periods of food scarcity (Kartzinel et al. 2019). Inter‐species competition for food resources impacts not only the survival and reproduction of the host but also affects the host's metabolism, immunological response, and behavior via selection pressure on the gut microbiome. A study on the human colon microbiome indicated that the gut microbiome sustains host health via symbiotic and competitive interactions among various species and influences the host's immune response (Flint et al. 2015). In addition, O'Toole and Cooney revealed how probiotics influence the gut microbiome through competitive mechanisms and enhance pigs and cows' adaptability by regulating metabolic functions (O'Toole and Cooney 2008). In herbivores, alterations in the gut microbiota during interspecies food competition are notably important. Investigations on pikas have shown that livestock grazing activities alter the species composition and abundance of grassland flora. These alterations impact not just the food supplies of herbivores but also their health and ability to adapt by modifying the composition of the gut microbiome (Li, Wang, et al. 2019). Research has demonstrated that food changes generated by grazing activities can selectively modify the makeup of the gut microbiome, further influencing the host's metabolism and immune response, thereby altering the competitive dynamics between species (Li, Li, et al. 2019). In the study of plateau zokors, it was found that through the adjustment of gut microbiota and the evolution of digestive tract morphology (such as the increase in cecal capacity), effective utilization of a high‐fiber diet was achieved. This indicates the importance of gut microbiota in the adaptation of rodents to extreme environments (Zhang et al. 2022). Studies indicate that the gut microbiota of rodents is affected by host species, diet structure, and geographical factors (Yang et al. 2024; Cao et al. 2024; Ren et al. 2025). Herbivorous rodents exhibit more gut microbiota diversity and possess an abundance of cellulose‐decomposing bacteria, while omnivorous rodents are characterized by microbial communities adept at degrading complex polysaccharides and proteins (Neha et al. 2025). These studies underscore the significance of gut microbiota for hosts in interspecies competition; gut microbiota may provide survival advantages for hosts.

**FIGURE 1:**
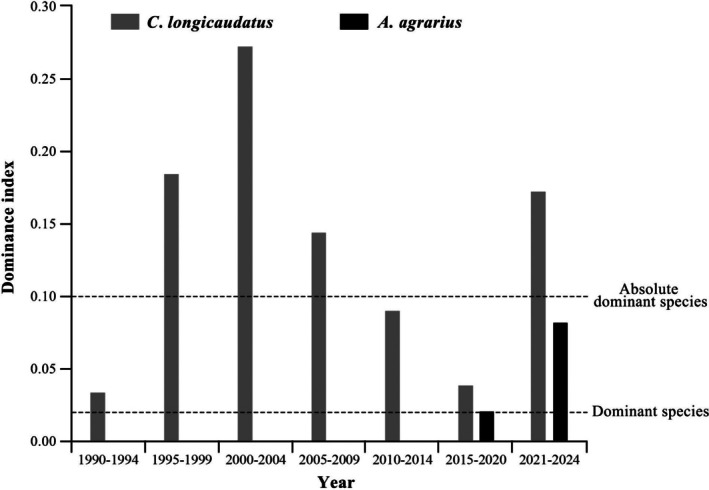
Species dominance histogram of *C. longicaudatus* and *A. agrarius* from 1900 to 2024. Dominant species were identified using the McNaughton Dominance Index (Y). The calculation formula: Y = Ni/N × fi. Where fi is the occurrence frequency of the i type, Ni is the number of individuals of the i type, and N is the total number of individuals in the same year. Y > 0.020 is the dominant species, Y > 0.10 is the absolute dominant species.

The long‐tailed dwarf hamster ( *Cricetulus longicaudatus* ) is mainly distributed in the eastern and central‐western regions of China, being the absolute dominant species in the farmland (Poplavskaya et al. 2018; Yang, Wang, et al. 2021). The striped field mouse ( *Apodemus agrarius* ) is among the most abundant and extensively dispersed rodents in the temperate zone of the palaearctic region (Wang et al. 2021). It is a dominant species in the Europe–Siberia and East Asia regions, the majority of which is located within China. This species is regarded as a prevalent rodent in the northeastern territories of Russia above 25° N latitude, in mainland China, and in the agricultural areas along eastern China (Koh et al. 1997). In Shanxi Province, *C. longicaudatus* and *A. agrarius* are co‐distributed species that exhibit interspecific competition. We systematically observed the population counts of *C. longicaudatus* and *A. agrarius* in Houyin Village, Xixian County, Shanxi Province from 1990 to 2024 and computed their dominance indices (Figure 1). The results revealed that from 1995 to 2009 and from 2021 to 2024, *C. longicaudatus* was the absolutely dominant species (Y > 0.10). Between 2010 and 2020, despite a sustained drop in the population of *C. longicaudatus* , it persisted as the predominant species in the region (Y > 0.02). The population of *A. agrarius* was consistently low from 1990 to 2014. Commencing in 2015, the population of *A. agrarius* observed steady growth, establishing itself as the predominant rodent species in the region (Y > 0.02), but it remained inferior in number compared to *C. longicaudatus* . The changes in the population sizes of the two species reflect the alterations in their survival adaptation to environment. The gut microbiota may play a crucial role in the adaptation of these two species. In the present study, we first compared the structure and diversity of gut microbiota between *C. longicaudatus* and *A. agrarius* , which coexist in the wild, using 16S rDNA sequencing techniques and explored the similarities and differences in the adaptive traits of these two species concerning food availability in their natural habitat. Moreover, by providing food from various nutritional categories to the two animals in a controlled laboratory environment, we elucidated the importance of gut microbiota in their dietary responses, analyzing the similarities and differences in their adaptability to food resources. Ultimately, by comparing analyses of the gut microbiomes of the two species, we investigate the dynamics of competition, adaptation, and survival within the same ecosystem. We propose that changes in diet will result in substantial modifications to the gut microbiota. We hypothesize that the gut microbiota will interact with the host and partake in interspecies and resource competition, and that the adaptive alteration of gut microbes offers an effective strategy for symbiosis between the two species amid interspecific competition.

## 2 Materials and Methods

### 2.1 Subjects and Experimental Design

In this study, we conducted two experiments.

#### 2.1.1 Design of Experiment One

First, we are investigating differences in the microbiome of species with distinct feeding strategies. The experimental design is outlined as follows: In the summer of 2024, to avoid any potential differences between the male and female, six samples each of healthy adult male *C. longicaudatus* and *A. agrarius* were obtained using cage catches in Houyin Village, Xixian County, Shanxi Province (36.729° N, 110.839° E). Rectal fecal samples were promptly collected in the field and preserved at −80°C until the 16S rDNA sequencing was conducted.

#### 2.1.2 Design of Experiment Two

We tested the adaptive alterations in gut microbiota of two species using an indoor experiment including the feeding of animals with food from various nutritional layers under differing dietary circumstances. The experimental design is outlined as follows: Healthy adult male *C. longicaudatus* and *A. agrarius* were collected in Huyan Village, Xixian County, Shanxi Province (36.729° N, 110.839° E). Following a two‐week acclimation in the laboratory, the experiment will commence. All animals are kept in individual cages under circumstances of 23°C ± 2°C temperature, 55% ± 2% relative humidity, a natural light cycle, and a sufficient diet. *C. longicaudatus* and *A. agrarius* were respectively divided at random into three groups according to their body mass: Con (Control, standard rodent diet, *n* = 6), HG (High‐fiber diet, *n* = 6), and HF (High‐fat diet, *n* = 6). Food composition is shown in Table 1. The animals will undergo continuous feeding for 28 days, with free access to water and food during the period. After 28 days, rectal feces from all experimental samples were collected and preserved at −80°C until 16S rDNA sequencing was conducted in the indoor experimental group. The experimental protocols were carried out strictly in compliance with the People's Republic of China's Ministry of Science and Technology's Regulations on the Administration of Laboratory Animals (SXAU‐EAW‐2024 M.JE.004022321).

**TABLE 1:** The components of standard food, high‐fat food, and high‐fiber food.

Content	Standard diet	High‐fat diet	High‐fiber diet
Crude fat (%)	6.2	21.4	3.9
Crude protein (%)	20.8	17.6	19.4
Neutral detergent fiber (%)	21.5	19.6	35.5
Acid detergent fiber (%)	12.5	10.6	21.4
Ash (%)	10.0	8.5	10.5
Caloric value (kJ/g)	17.5	19.7	17.3

### 2.2 DNA Extraction and 16S rRNA Gene Sequencing

Genomic DNA extraction from the total community was conducted utilizing an E.Z.N.A MagBind Soil DNA Kit (Omega, M5635‐02, USA), adhering to the manufacturer's guidelines. A Qubit 3.0 fluorescent quantitative analyzer was used to measure the library concentration, and 2% agarose gel electrophoresis was used to calculate the library size. Two universal bacterial 16S rDNA gene amplicon PCR primers (PAGE purified) were used: the amplicon PCR forward primer (5′‐CCTACGGGNGGCWGCAG‐3′) and amplicon PCR reverse primer (5′‐GACTACHVGGGTATCTAATCC‐3′) (designed and synthesized by Sangon BioTech, Shanghai). The reaction was set up as follows: microbial DNA (10 ng/μL) 2 μL; amplicon PCR forward primer (10 μM) 1 μL; amplicon PCR reverse primer (10 μM) 1 μL; and 2× Hieff Robust PCR Master Mix (Yeasen, 10105ES03, China) (total 30 μL). The plate was sealed and PCR was performed in a thermal instrument (Applied Biosystems 9700, USA) using the following program: one cycle of denaturing at 95°C for 3 min, first five cycles of denaturing at 95°C for 30 s, annealing at 45°C for 30 s, elongation at 72°C for 30 s, then 20 cycles of denaturing at 95°C for 30 s, annealing at 55°C for 30 s, elongation at 72°C for 30 s, and a final extension at 72°C for 5 min. All of the samples were then mixed together in a 1:1 ratio. Sangon BioTech received the samples and used a universal Illumina adapter and index to build a library. Prior to sequencing, a Qubit 4.0 Green double‐stranded DNA assay was used to measure each PCR product's DNA concentration, and a bioanalyzer was used to ensure quality control. And the information of quality filtering parameters is shown in the Table S1. After sequencing, the RDP database was used to taxonomically classify the representative sequences of bacterial OTU.

### 2.3 Bioinformatics Analysis

Following sequencing, the two brief Illumina reads were combined using PEAR software (version 0.9.8) based on their overlap, and the fastq files were processed to produce distinct FAST and QUAL files, which could subsequently be evaluated using standard methodologies. The effective tags were grouped into operational taxonomic units (OTUs) of ≥ 97% similarity using Usearch software (version 11.0.667). Chimeric sequences and singleton OTUs (containing only one read) were eliminated, following which the residual sequences were categorized into each sample according to the OTUs. The most abundant tag sequence was chosen as the typical sequence for each cluster. Bacterial and fungal OTU representative sequences were taxonomically identified by using BLAST searches against the RDP Database and the UNITE fungal ITS Database, respectively.

### 2.4 Data Analysis

The α‐diversity indices (including Chao1, Simpson, and Shannon indices) were quantified in terms of OTU richness. To assess sample adequacy, rarefaction curves of the observed numbers of OTUs were constructed, and all α‐diversity indices were calculated with Mothur software (version 3.8.31). The OTU rarefaction curve and rank abundance curves were plotted in R (version 3.6.0). To estimate the diversity of the microbial community of the sample, we compared the within‐sample (alpha) diversity by T‐test between two groups, and multiple group comparisons were made using analysis of variance (ANOVA) test. Beta diversity assesses the differences in microbiomes between samples. It calculates Jaccard and Bray‐Curtis distance matrices based on OTUs and combines them with dimensionality reduction methods such as principal coordinate analysis (PCoA) to obtain visual representations. These analyses were visualized using the Rvegan package (version 2.5‐6), and the distances between samples were ultimately presented in scatter plots to observe the differences in the structure of the intestinal microbiome communities. These analyses were visualized using the vegan package in R program (version 2.5‐6), and finally, the inter‐sample distances were presented as scatterplots. Venn diagrams were drawn using R 3.2.6 (venn diagram package) to describe common and gap flora between groups. Difference comparison is used to identify features with significantly different abundances between groups using LEfSe (version 1.1.0). The data were further examined, and network analysis was produced using R 3.2.6 and Gephi v.0.9.2 software; *p* < 0.05, |*R*| > 0.6 were considered statistically significant. Functional prediction analysis of bacteria and archaea using PICRUSt (v1.1.4) software, by comparing existing 16S rRNA gene sequencing data with a microbial reference genome database of known metabolic functions, enables the prediction of bacterial and archaeal metabolic functions. The experimental results were expressed as mean ± standard deviation (mean ± SD). SPSS 26.0 software was used to compare samples among groups by one‐way ANOVA. Statistical differences between treatments were considered significant at *p < 0.05, **p < 0.01, and ***p < 0.001.

## 3 Results

### 3.1 Experiment One

#### 3.1.1 Gut Microbiota Composition Between Two Species in Wild

**FIGURE 2:**
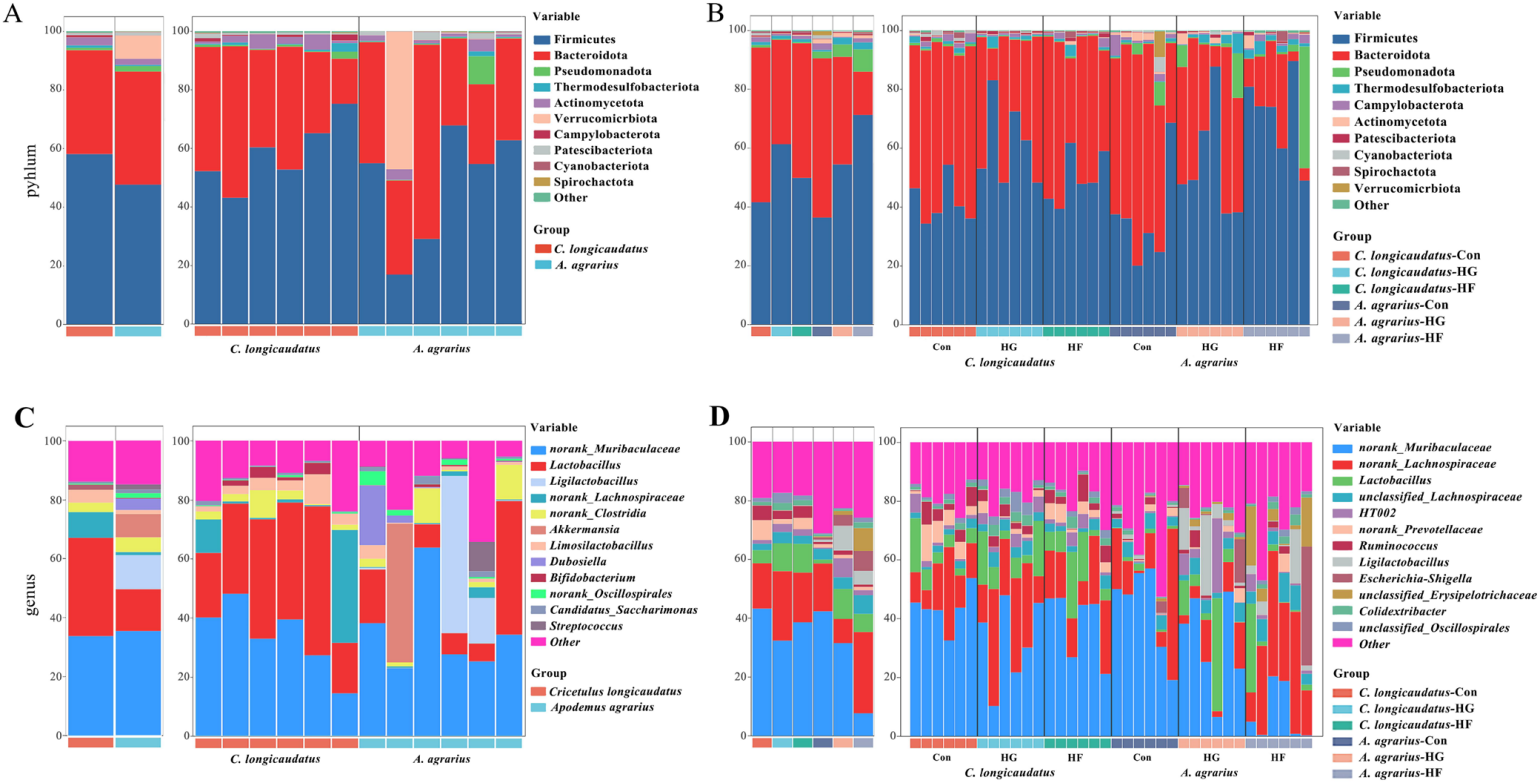
Composition of gut microbiota at phylum (A) and genus level (C) in wild *C. longicaudatus* and wild *A. agrarius* as well as *C. longicaudatus* and *A. agrarius* under different diets in the laboratory at phylum (B) and genus (D).

**FIGURE 3:**
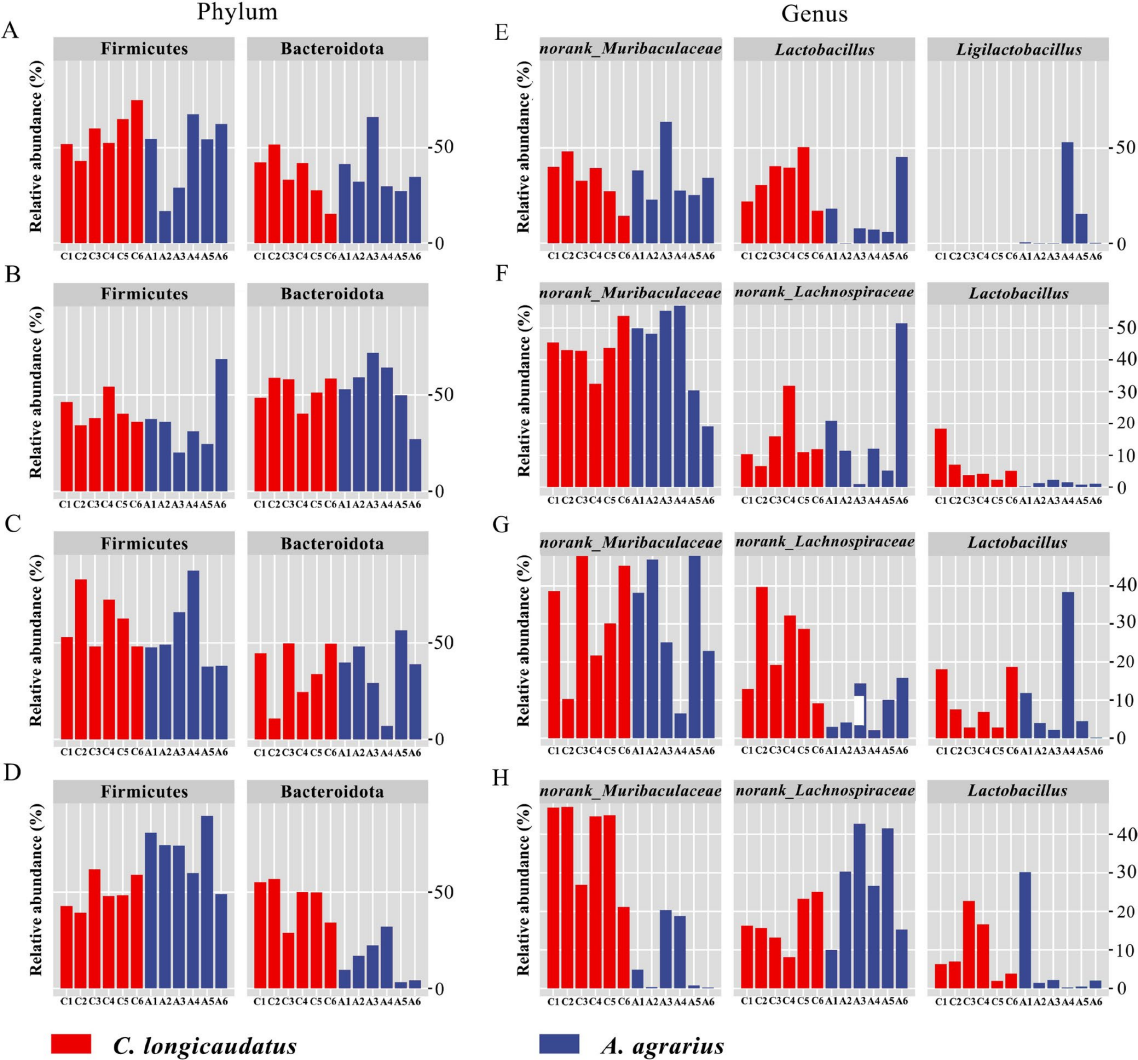
Analysis of dominant microbiota of wild *C. longicaudatus* and wild *A. agrarius* at phylum level (A) and at genus level (E), and *C. longicaudatus* and *A. agrarius* of Con group at phylum level (B) and at genus level (F), and *C. longicaudatus* and *A. agrarius* of HF group at phylum level (C) and at genus level (G), and *C. longicaudatus* and *A. agrarius* of HG group at phylum level (D) and at genus level (H).

In the first experiment, 1,085,196 valid sequences were obtained, cluster analysis was conducted on the sequences using a 97% similarity criterion, resulting in the identification of 4406 OTUs. Based on the OTU classification results, we conducted a phylum and genus‐level analysis of the gut microbiome composition of *C. longicaudatus* and *A. agrarius* (Figure 2A,C). There were no differences in the gut microbiota of the two wild species at the phylum level. At the genus level, the relative abundance of *Lactobacillus* in *C. longicaudatus* was significantly higher than that in *A. agrarius* . Moreover, our results indicate that the predominant phyla of the two wild species were Firmicutes and Bacteroidetes, while the most dominant genera of the two wild species were *norank_Muribaculaceae*, *Lactobacillus*, and *Ligilactobacillus*. Among them, the difference in the dominant gut microbiota between different individuals within *A. agrarius* in the field was greater than that of *C. longicaudatus* (Figure 3A,E).

#### 3.1.2 Gut Microbial Diversity and Differences Between Two Species in Wild

**FIGURE 4:**
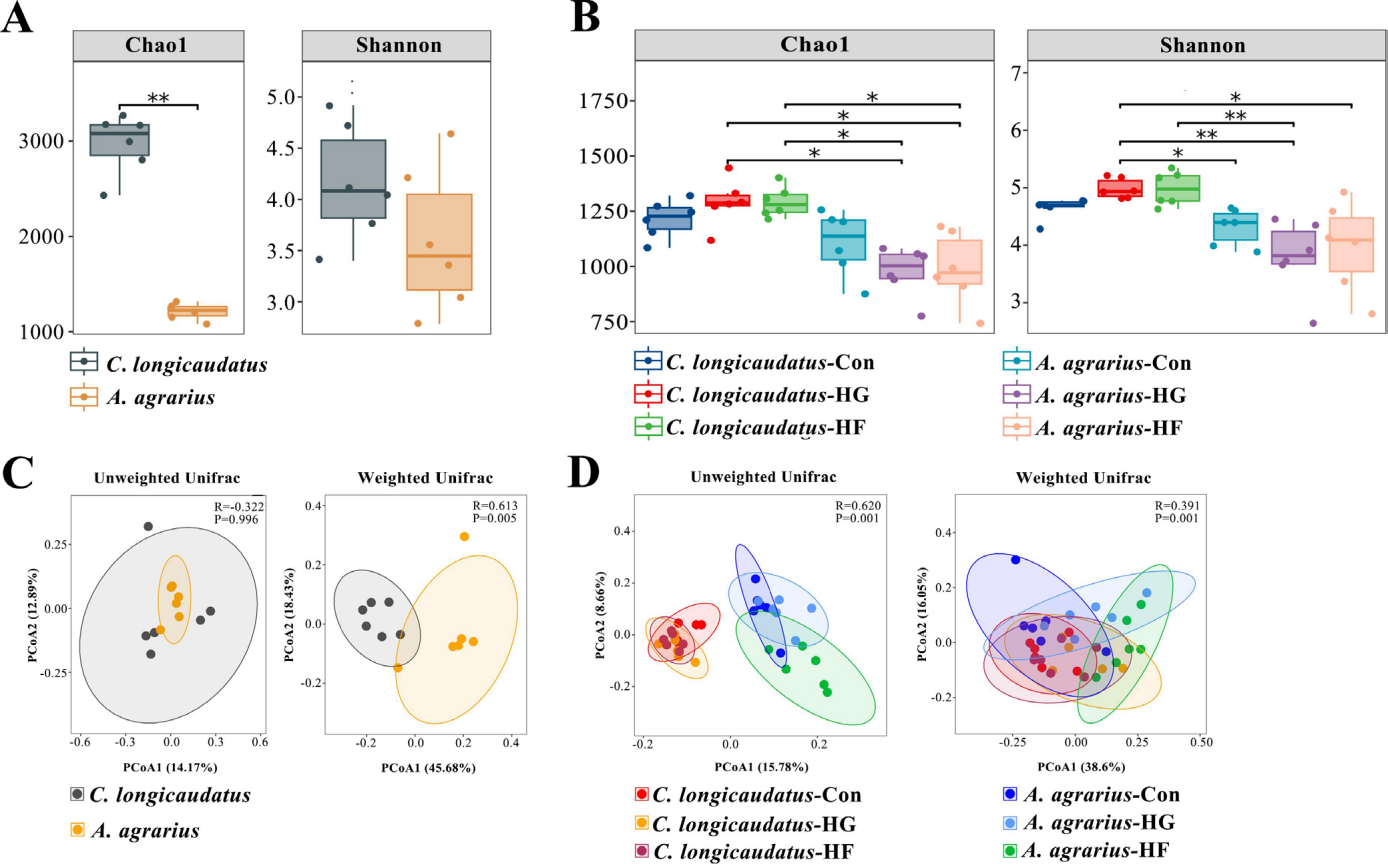
Diversity of microbiota of two species. Chao1 and Shannon index of two species in the wild (A) and under different diets in the laboratory (B). Unweighted UniFrac PCoA and Weighted UniFrac PCoA of two species in the wild (C) and under different diets in the laboratory (D).

**FIGURE 5:**
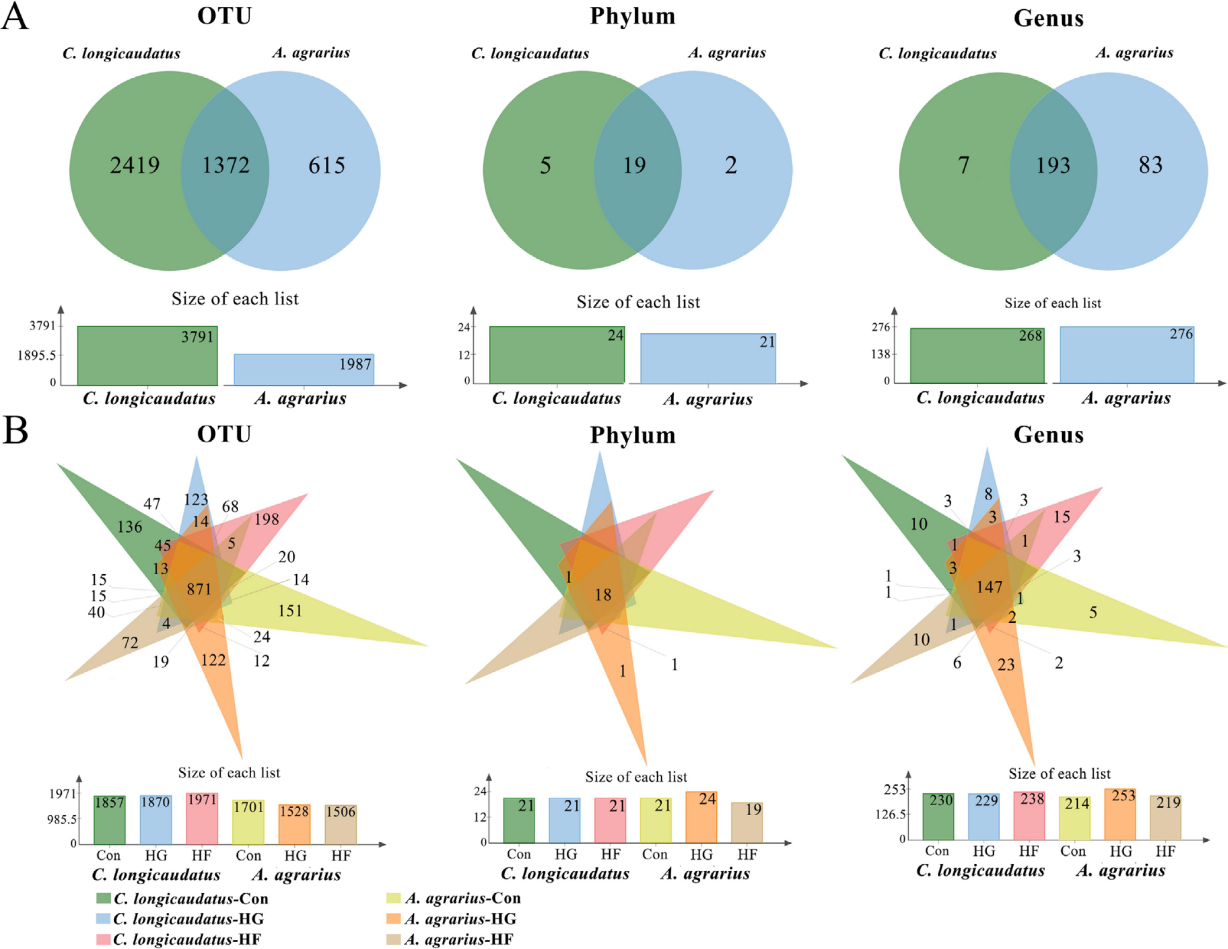
Veen diagram analysis of two species in the wild (A) and under different diet in laboratory (B).

**FIGURE 6:**
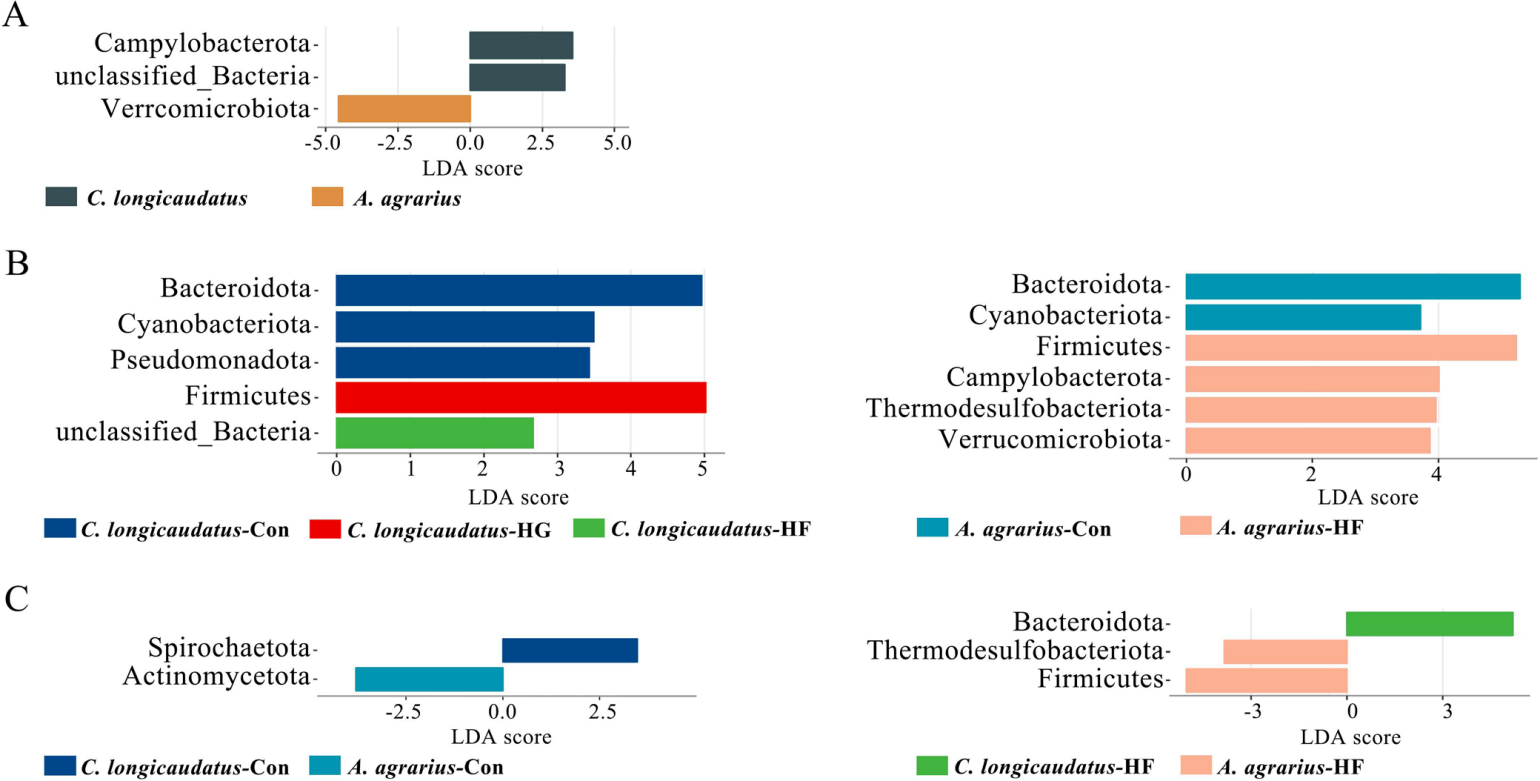
Analysis of gut microbiota difference between *C. longicaudatus* and *A. agrarius* . The different gut microbiota between two species in the wild (A). The different gut microbiota of *C. longicaudatus* and *A. agrarius* under different diets (B). The different gut microbiota between two species under the same diet (C).

The α‐diversity index (Chao1 and Shannon) of two wild species is shown in Figure 4A. There were similar trends in the Chao1 and Shannon indices. Among them, the Chao1 index of *C. longicaudatus* was significantly higher than that of *A. agrarius* , but there was no difference in the Shannon index between the two species. Firstly, through the weighted Unifrac PCoA analysis, the results showed that the gut microbial abundances between the two species in the wild were different (Figure 4C). Then, we used the LEfSe analysis on the differential microbiota between the two species; it was found that Campylobacterota and unclassified_Bacteria were markedly enriched in the gut of wild *C. longicaudatus* , whereas Verrcomicrobiota were significantly enriched in the intestines of wild *A. agrarius* (Figure 6A). Moreover, the unweighted Unifrac PCoA analysis showed that there was no difference in the β‐diversity of gut microorganisms between *C. longicaudatus* and *A. agrarius* in the wild (Figure 4C). Venn diagrams may explicitly illustrate the amount and proportion of each group of microbes (Figure 5A). The results indicated that the number of gut microbial in the phylum level of *C. longicaudatus* was considerably greater than that of *A. agrarius* . However, at the genus level, the gut microbial diversity of *A. agrarius* was higher than that of *C. longicaudatus*.

#### 3.1.3 Core Microbial Co‐Occurrence Networks of Two Species in Wild

**FIGURE 7:**
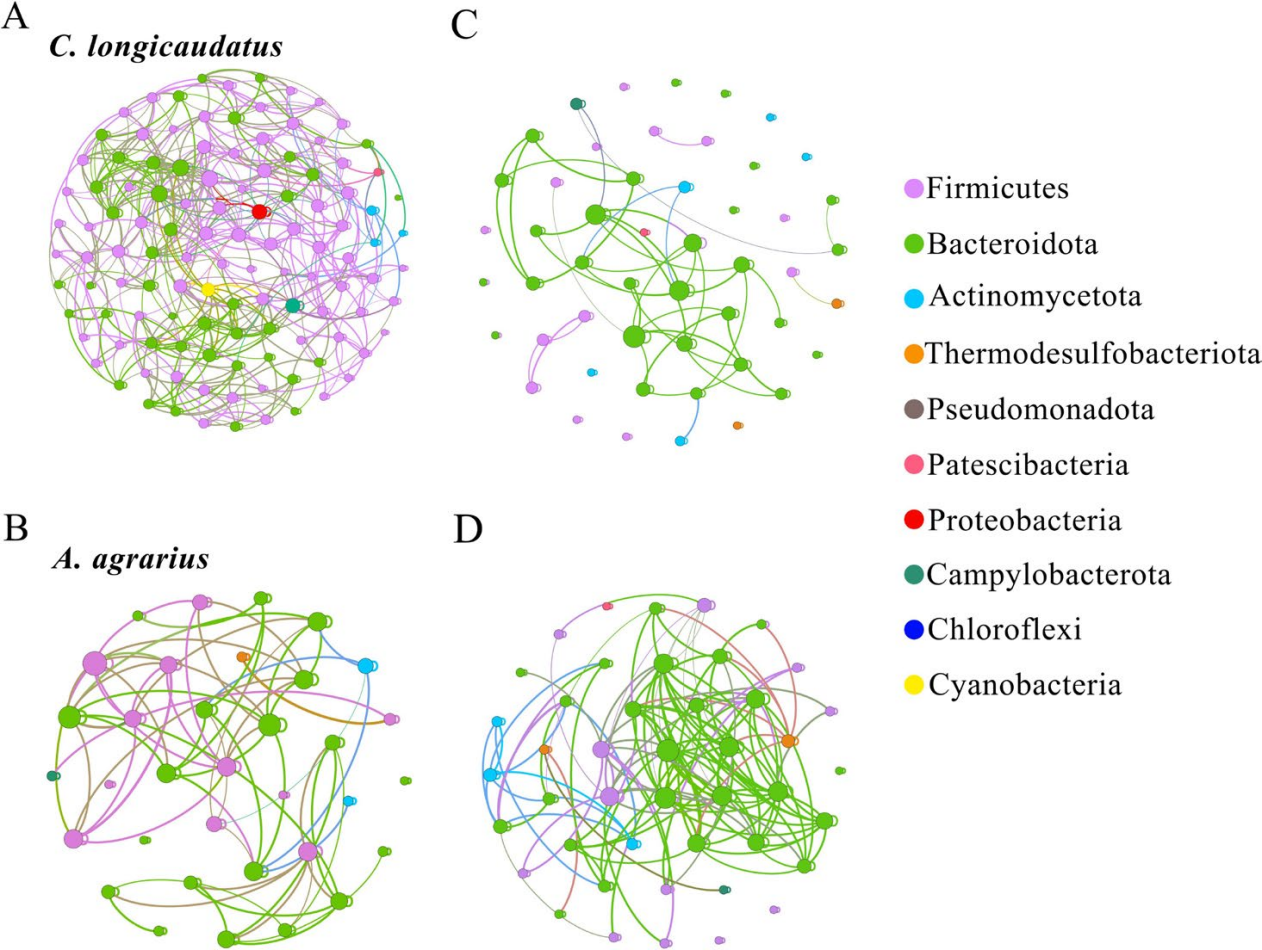
Co‐occurrence network analysis based on OTU. Co‐occurrence network of *C. longicaudatus* in the wild (A) and under three different food conditions in the indoor experiment (C) as well as the co‐occurrence network of *A. agrarius* in the wild (B) and under three different food conditions in the indoor experiment (D).

The co‐occurrence network analysis showed the OTU correlations within the gut microbiomes of wild *C. longicaudatus* and *A. agrarius* , with the top 2% of OTUs selected for each group. All samples exhibited OTUs with *R* > 0.6, *p* < 0.05, and abundance > 0.001 (Figure 7A,B). The wild *C. longicaudatus* had a very complex network of microbes, showing a strong connection rate of 71.81%, with 117 points and 596 links, and each point connecting to an average of 5.094 links. The points in the network are classified as Firmicutes, Bacteroidota, Actinomyceota, Patescibbacteria, Proteobacteria, Campylobacterota, and Cyanobacteria. The microbiological collinear positive correlation rate of wild *A. agrarius* was 99.67%, including 44 nodes and 175 edges, with each node averaging 3.977 edges. Network nodes were categorized as Firmicutes, Bacteroidota, Actinomyceota, and Thermodesulfobacteriota. Moreover, notable disparities in network structure were observed between the two species for topological properties, such as average degree, average path length, and density (Table 2).

**TABLE 2:** Network topology table on two experiments of *C. longicaudatus* and *A. agrarius* .

Group	Nodes	Edges	Positive edges (%)	Negative edges (%)	Average degree	Density	Diameter	Average path length	Average clustering coefficient	Modularity
Wild *C. longicaudatus*	117	56	71.81	28.19	10.188	0.088	8	3.315	0.603	1.441
*C.longicaudatus* in laboratory	50	92	95.65	4.35	3.680	0.075	6	2.946	0.588	0.701
Wild *A. agrarius*	34	98	99.67	0.33	5.765	0.175	7	2.984	0.613	0.613
*A. agrarius* in laboratory	44	175	94.86	5.14	7.955	0.185	6	2.592	0.681	0.453

*Note:* Nodes: Nodes represent microbial species. Edges: The edge represents the co‐occurrence relationship between the two species. Positive edges: The greater the positive edges, the closer the co‐occurrence relationship between the two species. Negative edges are opposite to positive edges. Average degree: The average number of edges connected to each node is expressed as the average degree. If the network graph is undirected, the calculation of the average degree is 2*edges/nodes. Density: A network with a higher density indicates that the co‐occurrence relationships among microorganisms are more complex and closely connected, which may reflect a higher stability of the microbial community; while a network with a lower density suggests that the co‐occurrence relationships among microorganisms are relatively fewer, and the community may be in a more loose state. Diameter: The maximum measurement length of the diameter of a network graph is the greatest value among the shortest distances between any two points, where each pair of points has one shortest distance. Average path length: The average distance between any two nodes in a network. It reflects the degree of separation between nodes in the network. Average clustering coefficient: The local clustering coefficient and the global clustering coefficient are parameters that reflect the close relationships among nodes in a network, and are also known as transitivity. Modularity: There may be some modules in the network, which are closely connected microbial communities. The microorganisms within a module are interrelated, while the connections between modules are relatively few.

### 3.2 Experiment Two

#### 3.2.1 Gut Microbiota Composition of Two Species With Different Food Fed in Laboratory

In the second experiment, 2,282,607 valid sequences were obtained. Cluster analysis of the sequences was conducted using a similarity criterion of 97%, resulting in the identification of 2959 OTUs. The three different feeding groups of the two species exhibited significant variations at both the phylum and genus levels, as well as notable variations in gut microbiota composition between the two species under same food conditions (Figure 2B). First, at the phylum level, the relative abundance of Firmicutes in the HG group of *C. longicaudatus* was markedly increased than that in Con group, however, the relative abundance of Bacteroidota considerably was decreased in the HG group. Unlike *C. longicaudatus* , the relative abundance of Firmicutes and Campylobacterota in the HG and HF groups of the *A. agrarius* was significant higher than that in Con group, but the relative abundance of Bacteriodota and Cyanobacteriota dramatically was decreased. Moreover, in comparison to the HG group, the relative abundance of Firmicutes markedly increased while Bacteriodota considerably dropped in the HF group. We also compared the gut microbiota composition between the two species under the same food conditions. First, the result showed that the relative abundance of Actinomycetota in *C. longicaudatus* was significantly higher than in *A. agrarius* under the Con condition. Furthermore, there was a considerable rise in the relative abundance of Firmicutes and a decrease in the relative abundance of Bacteroidota in *C. longicaudatus* under high‐fat diet conditions. Finally, there was no different in gut microbiota composition between two species under high‐fiber diet condition.

Additionally, there were various differences between the two species at the genus level (Figure 2D). In *C. longicaudatus* , the relative abundance of *Colidextribacter* and *unclassified_Oscillospirales* was significantly increased in the HG group compared to the Con group, while the relative abundance of *norank_Prevotellaceae* significantly decreased; the relative abundance of *unclassified_Oscillospirales* significantly decreased in the HF group compared to the HG group. In *C. longicaudatus* , the relative abundances of *Ligilactobacillus*, *unclassified_Lachnospiraceae*, *unclassified_Erysipelotrichaceae*, and *Colidextribacter* were significantly higher in the HG and HF groups than in the Con group, while the relative abundance of *norank_Muribaculaceae* was significantly decreased. Compared to the HG group, the relative abundance of *norank_Muribaculaceae* in *C. longicaudatus* was significantly increased in the HF group, while the relative abundances of *unclassified_Lachnospiraceae*, *norank_Lachnospiraceae*, *unclassified_Erysipelotrichaceae, Colidextribacter*, and *unclassified_Oscillospirales* significantly decreased. Moreover, under the same feeding conditions, the gut microbiota composition of the two species was also different at the genus level. First, under controlled food feeding conditions, there was no difference between the two species; however, under high‐fiber diet conditions, the relative abundance of *Ligilactobacillus* in *C. longicaudatus* was significantly greater than in *A. agrarius* , and the relative abundances of *norank_Lachnospiraceae*, *Colidextribacter*, and *unclassified_Oscillospirales* in *C. longicaudatus* were significantly lower than in *A. agrarius*. Under HF conditions, the relative abundances of *unclassified_Lachnospiraceae*, *unclassified_Erysipelotrichaceae*, and *unclassified_Oscillospirales* in *C. longicaudatus* were significantly higher than in *A. agrarius* , and the relative abundances of *norank_Muribaculaceae* and *Ruminococcus* in *C. longicaudatus* were significantly lower than in *A. agrarius* . Furthermore, our results indicate that there were more differences among different individuals in *A. agrarius* both at the phylum and genus levels (Figure 3). The dominant phyla at the phylum level were Firmicutes and Bacteroidetes (Figure 3B–D). At the genus level, the dominant genera were *norank_Muribaculaceae*, *norank_Lachnospiraceae*, and *Lactobacillus* (Figure 3F–H).

#### 3.2.2 Gut Microbiota Diversity of Two Species With Different Food Fed in Laboratory

Except that the gut microbiota diversity of *C. longicaudatus* in the control group was not different from that of *A. agrarius* , the gut microbiota diversity of *C. longicaudatus* was significantly greater than that of *A. agrarius* under high‐fat diet and high‐fiber diets (Figure 4B). Additionally, although there was no significant difference between the HG and HF groups of *C. longicaudatus* compared to the Con group, their diversity had relatively increased; in contrast, *A. agrarius* exhibited a decrease in diversity in the HG and HF groups compared to the Con group (Figure 4B).

Our results indicated that the analysis of unweighted Unifrac PCoA and weighted Unifrac PCoA demonstrated substantial differences between the two species (*p* = 0.001), with groups under varying dietary conditions within the same species clustering together (Figure 4D). The Venn diagram illustrates the quantity and proportion of microorganisms within each category (Figure 5B). Moreover, the results revealed that the numbers of gut microbiota of all groups of *C. longicaudatus* were surpassed by that of *A. agrarius* .

#### 3.2.3 The Different Microflora of Two Species With Different Food Fed in Laboratory

Further analysis of the differential microbiota between the two species utilizing LEfSe revealed that in *C. longicaudatus* , Bacteroidota, Cyanobacteriota, and Pseudomonadota were significantly enriched in the Con group, Firmicutes were significantly enriched in the HG group, and unclassified_Bacteria were significantly enriched in the HF group. In *A. agrarius* , Bacteroidota and Cyanobacteriota were significantly enriched in the Con group. Firmicutes, Cyanobacteriota, Thermodesulfobacteriota, and Verrucomicrobiota were significantly enriched in the HF group, but there was no significant enrichment of phylum in the HG group. Under controlled food feeding conditions, Spirochaetota was markedly enriched in *C. longicaudatus* , and Actinomycetota was notably enriched in *A. agrarius* . Under the high‐fat diets, Bacteroidota is significantly enriched in *C. longicaudatus* , and Thermodesulfobacteriota and Firmicutes are significantly enriched in *A. agrarius* , while no phylum exhibits significant enrichment under the high‐fiber diets (Figure 6B,C).

#### 3.2.4 Core Microbial Co‐Occurrence Networks of Two Species With Different Food Fed in Laboratory

The co‐occurrence network analysis showed the OTU correlations within the gut microbiomes of the two species, with the top 2% of OTUs selected for each group. All samples exhibited OTUs with *R* > 0.6, *p* < 0.05, and abundance > 0.001 (Figure 7C,D). In the core microbial co‐occurrence networks, each point represents a microbial species; the lines connecting two points represent the co‐occurrence relationship between the two species. The positive correlation reflects the strength of the co‐occurrence relationship between the two points. The greater the positive correlation, the closer the co‐occurrence relationship between the two species is. The microbial co‐occurrence network structure in *C. longicaudatus* was less intricate than that in *A. agrarius* , exhibiting a positive correlation rate of 95.65%, comprising 34 nodes and 98 edges, with an average of 2.882 edges per node. The network nodes were categorized as Firmicutes, Bacteroidota, Actinomyceota, Thermodesulfobacteriota, and Patescibbacteria. The co‐occurrence network in *A. agrarius* had a positive correlation rate of 94.86%. The network had 50 nodes and 92 edges, with each node exhibiting an average of 1.84 edges. The nodes were categorized as Firmicutes, Bacteroidota, Actinomyceota, Thermodesulfobacteriota, and Campylobacterota. Moreover, there were notable variations in network structure between the two species for topological properties, such as average degree, average path length, and density.

### 3.3 Comparative Analysis of Gut Microbiota of the Two Species in the Field and Fed Different Foods in the Laboratory

#### 3.3.1 Comparative Analysis of Gut Microbiota Composition

Results showed that the dominant phylum both in field and indoor experiments of the two species was Firmicutes and Bacteroidota. At the genus level, the dominant genus in the field was *norank_Muribaculaceae*, *Lactobacillus*, and *Ligilactobacillus*, while the dominant genus in the indoor experimental group was *norank_Muribaculaceae*, *norank_Lachnospiraceae*, and *Lactobacillus*. Compared to indoor experiments, the Firmicutes/Bacteroidetes (F/B) ratio of *C. longicaudatus* in the wild was decreased, but the F/B ratio of *A. agrarius* in the wild was significantly increased. Nonetheless, there were no substantial alterations in the microbiome composition across the two experiments, despite observable variations in bacterial abundance (Figure 2).

#### 3.3.2 Comparative Analysis of Gut Microbiota Diversity and Differences

The trends in the Chao1 and Shannon indices were consistent; *C. longicaudatus* exhibited a higher α‐diversity level compared to *A. agrarius* , both in field and laboratory experiments. The α‐diversity of the wild *C. longicaudatus* was significantly increased compared to the laboratory, but the α‐diversity of *A. agrarius* exhibited no variation among the wild and laboratory experiments. The results of the Venn diagram were consistent with the α‐diversity results. The gut microbiome number of the wild *C. longicaudatus* and the wild *A. agrarius* was higher than that observed in the laboratory experiments; however, the difference for *A. agrarius* between the two experiments was not significant (Figure 5). The LEfSe analysis results indicated that both in field and laboratory experiments showed considerable enrichment of Campylobacterota and Bacteroidota in *C. longicaudatus* , while Verrcomicrobiota was highly enriched in *A. agrarius* (Figure 6).

#### 3.3.3 Analysis of Core Microbial Co‐Occurrence Network

The microbial co‐occurrence networks of the two species in field and laboratory experiments showed significant differences. Among them, the microbial co‐occurrence network of the wild *C. longicaudatus* was the most complex, while that in laboratory experiments was the simplest. In contrast, the microbial co‐occurrence network of the wild *A. agrarius* was simpler than that in the laboratory experiment, which is the opposite of *C. longicaudatus* (Figure 7). Moreover, notable disparities in network structure were observed between the two experiments for topological properties, such as average degree, average path length, and density (Table 2).

#### 3.3.4 Analysis of the Differences in Gut Microbiota Metabolic Functions

**FIGURE 8:**
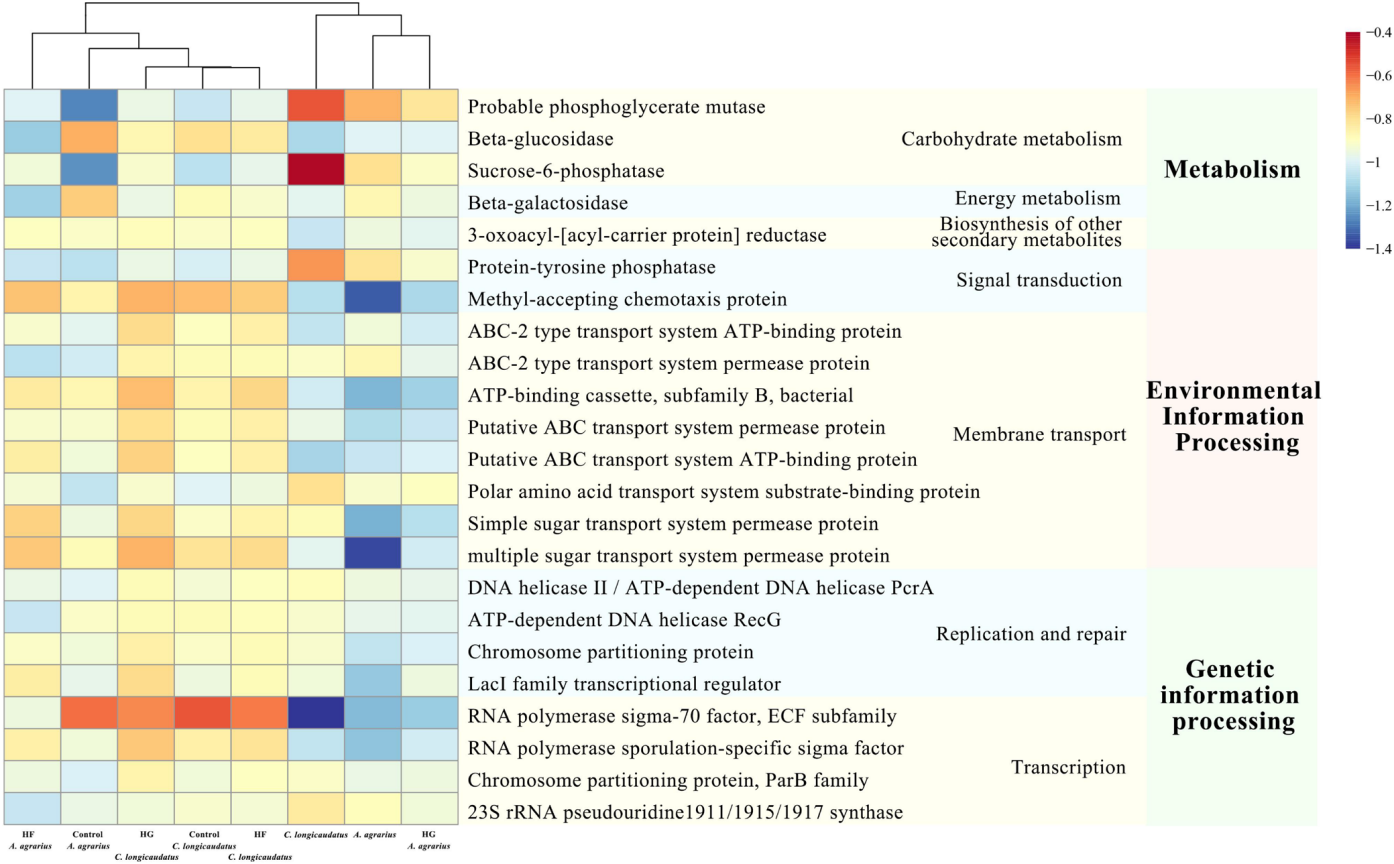
Functional analysis of gut microbiota of *C. longicaudatus* and *A. agrarius* in the wild and under the different diet.

The results showed that the variational metabolic pathways include carbohydrate metabolism, energy metabolism, the biosynthesis of additional secondary metabolites, signal transduction, transmembrane transport, and transcription (Figure 8). Within these metabolic processes, the metabolic functions of the two species in their natural habitat exhibit notable differences, including likely phosphoglycerate mutase, sucrose‐6‐phosphatase, methyl‐accepting chemotaxis protein, multiple sugar transport system permease protein, and RNA polymerase sigma‐70 factor.

## 4 Discussion

The adaptability of gut microbiota provides an effective strategy for sympatric proximal species to coexist in interspecific competition. Research indicates that variations in the responses of gut microbiomes across different populations to interspecies competition might influence their development and survival (He et al. 2018). The community composition of gut microbiomes in rodents arises from intricate interactions between bacteria and their hosts (Tao et al. 2024). Nevertheless, many extrinsic factors also affect the composition and diversity of gut microbiomes (Van Leeuwen et al. 2020). That complex and interrelated environmental variables influencing gut microbiome composition suggest that gut microbiomes have either a direct or indirect effect on the ecological problems and environmental stresses encountered by organisms (Coyte and Rakoff‐Nahoum 2019).

The *C. longicaudatus* has a rather limited global distribution (Poplavskaya et al. 2018; Yang, Wang, et al. 2021), but the *A. agrarius* is extensively dispersed globally (Wang et al. 2021). According to our early field results, since 1990, the *C. longicaudatus* has been the dominant rodent species in Shanxi province, while the *A. agrarius* has only recently become the dominant rodent species in the province. Although its dominance is lower than that of the *C. longicaudatus* , it still shows a trend of continuous increase. It has been suggested that animals migrating between different environments either exhibit changes in their gut microbiota to be similar to the local species or that the functions of their gut microbiota predictably converge with those of the species in the new habitat (Bletz et al. 2016). In the present study, the results showed that there was no significant difference between the two species at the phylum level in the wild. But our results revealed significant differences in the gut microbiota of wild *C. longicaudatus* and *A. agrarius* at the genus level, with wild *C. longicaudatus* exhibiting a greater abundance of *Lactobacillus*. *Lactobacillus* has fermentation and the ability to inhibit pathogenic bacteria, which can help *C. longicaudatus* improve plant digestion and resist pathogens, and enhance nutrient absorption and immune function (Slover and Danziger 2008; Calasso and Gobbetti 2011; Pot et al. 2014), providing a competitive advantage for *C. longicaudatus* in the natural environment. Moreover, Campylobacterota and unclassified_Bacteria were significantly enriched in the intestines of the *C. longicaudatus* , while Verrcomicrobiota was significantly enriched in the intestines of the *A. agrarius* . However, the predicted functions of the gut microbiota in the two species were different, which was in contradiction with the results of previous studies. Furthermore, the results of this study showed that there were greater differences among individuals of *A. agrarius* in terms of microbial composition and diversity than *C. longicaudatus* . Research has shown that microbial taxon similarity is higher in hosts living in groups with high population densities and frequent contact between individuals (Amato 2013). For example, cohabiting, genetically unrelated partners have more similar gut microbiota communities than individuals living in different households (Yatsunenko et al. 2012; Song et al. 2013; Mosites et al. 2017). Our long‐term field studies of rodent trapping indicated that *C. longicaudatus* predominantly reside in agricultural shrubs and exhibit a higher population density, whereas *A. agrarius* inhabit forest grasslands characterized by more intricate terrain. Therefore, the degree of interaction between *C. longicaudatus* individuals is relatively high, while the degree of interaction between *A. agrarius* individuals is relatively low. This might be the reason for the significant variations in the gut microbiota among individuals of *A. agrarius* . Moreover, our results revealed that the microbiota diversity of the *C. longicaudatus* was significantly higher than that of the *A. agrarius*. Increased microbial diversity correlates with improved immunological response and nutrient absorption in the host (Ross et al. 2024), which might give *C. longicaudatus* a competitive advantage in the wild by enabling the utilization of diverse food sources. Co‐occurrence network analysis also indicated that the microbial network of *C. longicaudatus* was more complex in wild environments. This may indicate that their gut microbiome is more stable, which improves their health in competitive environments. This corroborates previous studies indicating that complex networks may enhance the microbiome's resistance to disturbances (Kajihara and Hynson 2024). Although the network structure of *A. agrarius* was simpler than that of *C. longicaudatus* , the interaction ability between the microbiota of *A. agrarius* was stronger than that of *C. longicaudatus* . Therefore, we hypothesize that *A. agrarius* has a very strong ability of microbiota coordination to adapt to the environment. Moreover, the prediction of the metabolic functions of the gut microbiota indicates that there are significant differences in pathways such as carbohydrate metabolism and energy metabolism between the two species, with *C. longicaudatus* exhibiting increased activity for probable phosphoglycerate mutase and sucrose‐6‐phosphatase. Research indicates that metabolic activity may correlate with nutrient uptake capacity, providing an energetic advantage in competition through carbohydrate metabolism (Hornung et al. 2018), while variations in metabolic function may signify reliance on particular resources, aligning with the theory of nutritional competition (Valdes et al. 2018). This indicates that the metabolic advantage of *C. longicaudatus* may enhance its competitive standing by effective energy extraction, but the metabolic limitations of *A. agrarius* may restrict its resource use efficiency.

What accounts for the gut microbiota differences? Primarily, dietary variations are the main factor. In the present study, we subjected the two species to different food diets in the laboratory. The results indicated that there were varying degrees of changes in the composition and diversity of their gut microbiota, which was similar to previous results (Cao et al. 2024). However, when the two species were exposed to the same food, the composition of the gut microbiota of the two species was similar, but the abundance of microbiota was different. First, the relative abundance of Actinomycetota was significantly higher in *C. longicaudatus* compared to *A. agrarius* under the control diet, and Actinomycetota is crucial for the breakdown of cellulose and lignin (Heurich et al. 2015; Lewin et al. 2016). Second, under a high‐fat diet, *A. agrarius* exhibited a significantly higher relative abundance of Firmicutes and Campylobacterota as well as a significantly lower relative abundance of Bacteriodota and Cyanobacteriota. *C. longicaudatus* exhibited similar trends of changes with *A. agrarius* , as Bacteriodota increased and Firmicutes decreased, but there were no differences between HF group and Con groups. Moreover, under a high‐fat diet, *C. longicaudatus* exhibited a significantly higher level of Firmicutes and a significantly lower level of Bacteroidota compared to *A. agrarius* . Finally, under a high‐fiber diet, *C. longicaudatus* exhibited a significantly higher relative abundance of Firmicutes and a significantly lower relative abundance of Bacteriodota than Con groups. While *A. agrarius* exhibited a similar trend of change with its HF group. And *A. agrarius* had a high level of Bacteroidota and a low level of Firmicutes compared to its HF group. However, there were no differences between *C. longicaudatus* and *A. agrarius* under the high‐fiber diet. Firmicutes may break down fiber and cellulose to volatile fatty acids (Hu et al. 2017; Thoetkiattikul et al. 2013), and Bacteroidota can increase the decomposition of carbs and proteins and boost the nutrition utilization rate of the host (Waite and Taylor 2014). Furthermore, they all significantly contribute to the enhancement of host immunity and the maintenance of gut microbial balance (Sears 2005). It indicates that the two species adapt to different foods by regulating different microbiota.

At the genus level, compared to the Con group, the *C. longicaudatus* in a high‐fiber diet exhibited an increased relative abundance of *Colidextribacter* and *unclassified_Oscillospirales*, and a decreased relative abundance of *norank_Prevotellaceae*. In the high‐fat and high‐fiber diet, *A. agrarius* exhibited a significant increase in the relative abundance of *Ligilactobacillus*, *unclassified_Lachnospiraceae*, *unclassified Erysipelotrichaceae*, and *Colidextribacter*, and a decrease in the relative abundance of *norank_Muribaculaceae*. Under the same dietary conditions, the two species also showed differences at the genus level. Under the high‐fiber diet, *C. longicaudatus* has a higher relative abundance of *Ligilactobacillus* and a lower relative abundance of *norank_Lachnospiraceae*, *Colidextribacter*, and *unclassified_Oscillospirales* than *A. agrarius* . Under the high‐fat diet, *C. longicaudatus* exhibited a significantly higher relative abundance of *unclassified_Lachnospiraceae*, *unclassified_Erysipelotrichaceae*, and *unclassified_Oscillospirales* and a lower relative abundance of *norank_Muribaculaceae* and *Ruminococcus* than *A. agrarius. Colidextribacter* may participate in carbohydrate metabolism, the generation of short‐chain fatty acids (SCFAs), and the ecological equilibrium of the gut microbiota (Ricaboni et al. 2016; Zhao et al. 2023), and *unclassified_Oscillospirales* plays an auxiliary or synergistic function within the ecological network of gut microbiota (Gophna et al. 2017; Yang, Li, et al. 2021). The abundance of *Prevotellaceae* is significantly associated with plant‐based diets, such as those rich in fiber and carbohydrates (Erejuwa et al. 2014). These alterations may have enhanced the utilization of high‐fiber foods in *C. longicaudatus* , thereby providing supplementary energy to help it survive in the interspecific competition. *A. agrarius* has a lower adaptability to a high‐fiber diet. Although an increase of *Ligilactobacillus*, *unclassified_Lachnospiraceae*, *unclassified_Erysipelotrichaceae*, and *Colidextribacter* may correlate with their fiber metabolism (Turnbaugh et al. 2008; Ricaboni et al. 2016; Guerrero Sanchez et al. 2022; Kim et al. 2022), the effect is limited. Moreover, the degree of difference between the two species under a high‐fiber diet was lower than those under a high‐fat diet. These differences highlight that *C. longicaudatus* and *A. agrarius* have different eating habits, indicating that *A. agrarius* might be better suited for a high‐fat diet rather than a high‐fiber diet.

Furthermore, *C. longicaudatus* demonstrated an increase in gut microbiota diversity when given a high‐fat and high‐fiber diet, indicating superior adaptability to dietary modifications. This finding supports previous studies indicating that high‐fat and high‐fiber diets may improve metabolic flexibility by promoting beneficial microbes such as Firmicutes (Heiman and Greenway 2016). The gut microbiota diversity in rodents correlates with their ecological strategy (Maurice et al. 2015; Anders et al. 2021; Zhao et al. 2024). The lower gut microbiota diversity of *A. agrarius* may indicate that its niche adaptability is limited. The notable variation in β‐diversity reflected a divergence in microbiota structure between the two species, potentially linked to dietary factors and competitive pressures, aligning with prior research findings (Senghor et al. 2018; Pan et al. 2023). Additionally, the Venn diagram showed that the microbiota in *C. longicaudatus* was richer, which may give it a wider range of ecological functions. The analysis of microbiota differences indicated that the enrichment of Firmicutes in the HG group of *C. longicaudatus* was closely related to the high lipid metabolic requirement, which supported the energy requirement (Lee 2013). Unclassified Bacteria in the HF group reflect the adaptability of unknown‐function bacteria (Jeppe et al. 2022). The enrichment of Thermodesulfobacteriota in the HF group of *A. agrarius* might be related to its adaptability to an extreme environment (Montecillo 2024), but the results indicated the competitive ability of *A. agrarius* was still limited.

The gut microbial collinear network diagrams exhibited greater variation between the two species, both in natural habitats and controlled environments. Wild *C. longicaudatus* showed a more intricate network with a relatively low positive correlation rate; however, after being fed various diets, the network structure was simplified, and the positive correlation rate increased. This change likely resulted from the reduction of microbial interaction complexity due to a single diet, contrasting with previous research, and indicating that dietary balance significantly impacted the network's structure (Sun et al. 2023). Conversely, wild *A. agrarius* presented a simple network with a relatively high positive correlation rate; after exposure to different diets, the network structure became more complex, accompanied by a decrease in the positive correlation rate. These results indicate that *A. agrarius* is adapting to dietary changes (Luo et al. 2024). We hypothesize that *C. longicaudatus* may provide it a competitive advantage by improving microbiota stability, while the network modification in *A. agrarius* may indicate short‐term adaptation. The predicted functions of the gut microbiomes in the two rodent species align. Indoor experiments exhibit notable distinctions when contrasted with field experiments. Under different diets, *C. longicaudatus* can adjust its gut microbiota better than *A. agrarius* , but the number of microbiota in *C. longicaudatus* in the laboratory had decreased compared to that in the wild, while *A. agrarius* in the laboratory had more microbiota than that in the wild. These results may indicate that *C. longicaudatus* survived well by using its gut microbiota to adapt to its habitat environment, but *A. agrarius* had a different survival strategy, which makes it less adaptable and resourceful. Therefore, the population number of *A. agrarius* is consistently lower than that of *C. longicaudatus* . It is possible that due to changes in vegetation or the types of crops being planted, and because *A. agrarius* is gradually adapting to the environment and changing its way of life, its population number is showing an upward trend.

## 5 Conclusion

In the present study, 16SrDNA was used to investigate the gut microbial composition, diversity, co‐occurrence networks, and metabolic functions of the *C. longicaudatus* and *A. agrarius* , which are two species distributed in the same domain, under natural ambient and varying dietary situations. Our data revealed that there were significant differences in gut microbial structure and diversity between the two species, both in the wild and during varying dietary situations. Specifically, the *C. longicaudatus* demonstrated high alpha diversity and abundance of *Lactobacillus*, whereas *A. agrarius* showed substantial enrichment of Verrucomicrobiota. Additionally, the degree of interaction among wild *C. longicaudatus* individuals was relatively high, while the degree of interaction among wild *A. agrarius* individuals was relatively low. Moreover, wild *C. longicaudatus* had a more complex co‐occurrence network with a low level of positive correlation rate; however, after being fed various diets, the network structure was simplified, and the positive correlation rate increased. On the contrary, wild *A. agrarius* had a simple co‐occurrence network with a high level of positive correlation rate; after exposure to different diets, the network structure became more complex, accompanied by a decrease in the positive correlation rate. Our results also revealed differences in dietary adaptation between the two species. *C. longicaudatus* exhibited greater microbial adaptability under high‐fat and high‐fiber dietary conditions than *A. agrarius* , as indicated by a significant rise in the Firmicutes/Bacteroidetes ratio. While *A. agrarius* demonstrated reduced adaptation to dietary changes, it had a stronger ability to adapt to high‐fat food than to high‐fiber food. These data revealed that *C. longicaudatus* employed microbial diversity for dietary adaptability, whereas *A. agrarius* relied on microbial interactions for rapid adaptation, indicating that the two species employed distinct ecological adaptation strategies. Furthermore, *C. longicaudatus* had relatively high adaptability to different foods, while *A. agrarius* had less adaptability to food resources than *C. longicaudatus* , which may be one of the reasons why *A. agrarius* is less successful than *C. longicaudatus* . Finally, our data revealed significant alterations in carbohydrate metabolism pathways, such as sucrose‐6‐phosphatase, between the two species in the wild, while, after varying dietary situations, there was no significant change in the functional prediction. Our research provides new evidence supporting the view that the number and diversity of gut microbiota change flexibly, alleviating the competition for resources between two species of rodents that are distributed in the same area and providing them with an effective survival strategy.

## Author Contributions

**Yue Ren:** formal analysis (lead), funding acquisition (lead), investigation (lead), methodology (lead), supervision (lead), writing – review and editing (lead). **Mengfan Tao:** data curation (lead), formal analysis (lead), investigation (lead), writing – original draft (lead), writing – review and editing (lead). **Guangtong Guo:** data curation (equal), software (equal). **Kuiyou Chen:** data curation (equal), software (equal). **Xinsheng Pu:** investigation (equal), software (equal). **Yu Hou:** investigation (equal). **Xin'gen Yang:** funding acquisition (lead), methodology (lead), project administration (lead).

## Ethics Statement

All animal operations follow the guidelines established by the Animal Care and Use Committee of Shanxi Agricultural University's College of Veterinary Medicine. The committee approved this study.

## Conflicts of Interest

The authors declare no conflicts of interest.

## Supporting information


**Table S1:** Quality filtering parameters.

## Data Availability

16S rDNA sequences data accessibility: https://doi.org/10.5061/dryad.mgqnk99b4
